# Supplementary material for: Chromothripsis during telomere crisis is independent of NHEJ, and consistent with a replicative origin
Source: Genome Res. 2019 May;29(5):737–49. doi: 10.1101/gr.240705.118 (PMC6499312; doi:10.1101/gr.240705.118)
Supplement: Supplemental Material [file supp_gr.240705.118_Supplemental_file_1.zip › contigs/annotated_contigs/DB94/contig.4.DB94_length_810_mean_cov_7.84197530864.docx]

**DB94_length_810_mean_cov_7.84197530864**

```
GGTCTGCTCCAAAGACTCTGATTTTTTAATGTTTAATTTTTATTTTTAATTGACAACAAATAGAATTGCCATATAGATATAGCAATGCC
 >chr2:137435864-137436080 + E=5e-112 p=0e+00
ACTTCTATATATATAATATACATATATATCAAATACATATTATATATATAATATACATATATATCAAATACATATTATATATAATATAC

ATATATATCAAATACATATTATATATAATATACATA|TATAA|TACCCATTTATATATATATATATATATA|AA|TTCTGTTTACTCTG
                                          >chr5:27346795-27346823 - E=5e-02 p=4e-02
GTCCCTAGAGTAAACAGAGTTCCAATCTGTGAACATTAAGGGCATTTTTAGTTATACTTCATGTATTTTAGTATACAGTTCCATTCTTC
>chr2:137436885-137437181 - E=3e-166 p=4e-02
TTAAATTTGCCTCACTAGTCTCTTGAGTAACAGATTTATATTGAATCATATATGTGTAGAATTATGTCACTATAATAATATGCGATATA

ATAAACCTATAAACATGTAGGTAGTAAATGATACTTATTATTGTAATTATATAATCTATAGAAGTGTAAGTTGATATCCCAGCACACCT

CTAAACACTCCAAA|A|TATATATATATATATATAAATTTTTATATATATATAAAAATTGACACCAGTATCCCGAAAAGACATATGCCC
               >chr2:137436162-137436416 + E=4e-127
TGTCATGTTCATTGCAGTATTATTCACAATATCTAACATATGGAATCAACCTAAGTGTCCATCGGTTGATGAATGTATTTTAAAATGTG

GCGTATATACACAGTAGAATACTATTCAGCCTTAAAAAAAGAAAGGAATTCTGTTATTTGCAACAACATGAATAAACATAGAGGGTATT

ATGCTAAGTAAAATA
```
